# Supplementary figures and images for: The ultrastructure of infectious L-type bovine spongiform encephalopathy prions constrains molecular models
Source: PLoS Pathog. 2021 Jun 1;17(6):e1009628. doi: 10.1371/journal.ppat.1009628 (PMC8195424; doi:10.1371/journal.ppat.1009628)

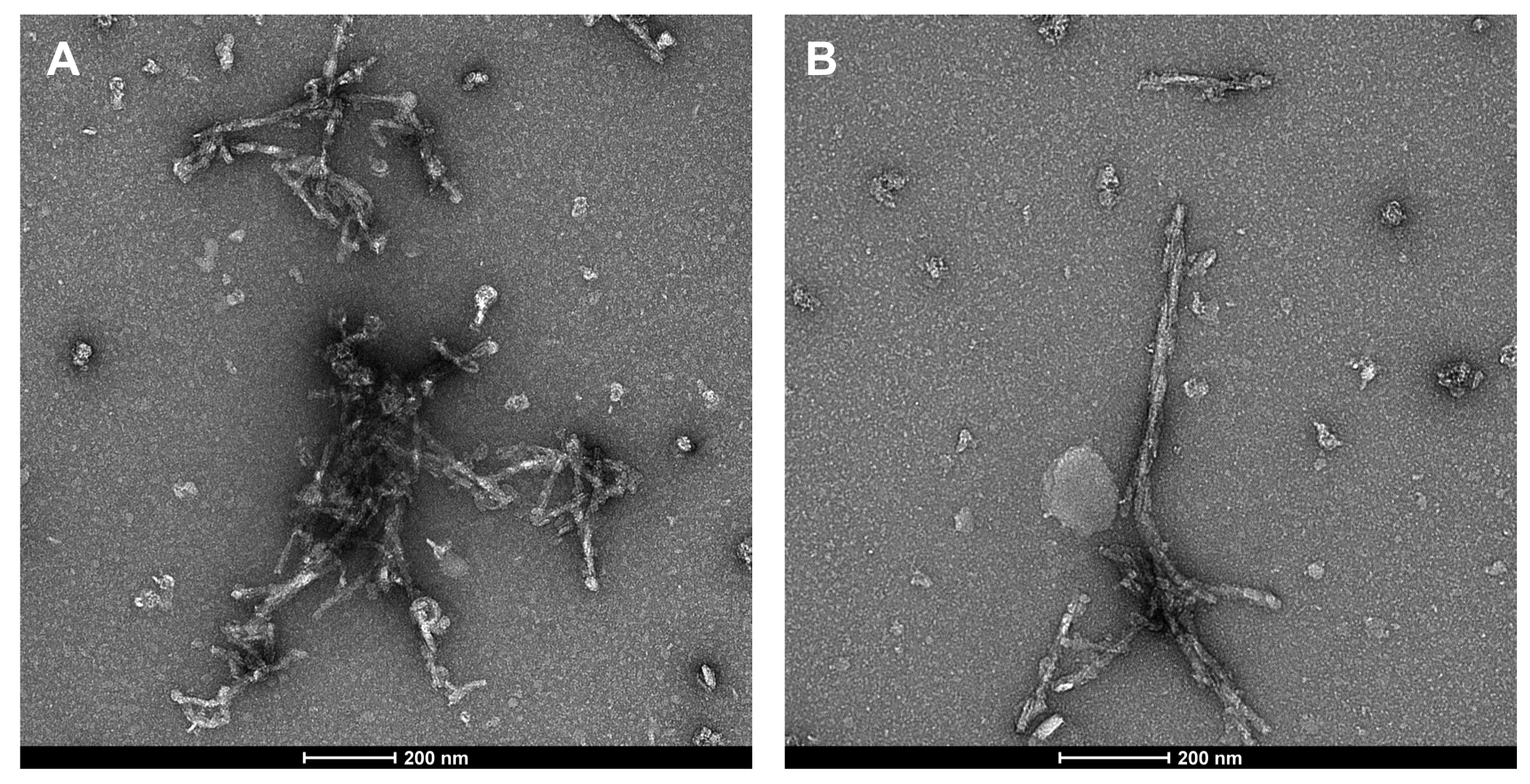

Supplement: S1 Fig — The pellet 1 sample was taken before the sucrose step gradient ultracentrifugation. (TIF) [file ppat.1009628.s001.tif]
