# Supplementary material for: The ultrastructure of infectious L-type bovine spongiform encephalopathy prions constrains molecular models
Source: PLoS Pathog. 2021 Jun 1;17(6):e1009628. doi: 10.1371/journal.ppat.1009628 (PMC8195424; doi:10.1371/journal.ppat.1009628)
Supplement: Attachment — Submitted filename: review.pdf [file ppat.1009628.s002.pdf]

The manuscript describes structural studies of a specific L-type strain of infectious prions. Structural studies of brain-derived prions are extremely complicated, so our knowledge on the structure of infectious prions is still limited and each new piece of information is important. So, I strongly support publication of this paper in PLoS Pathogens.

The experiments are well-executed, and the paper is well-written, so I have just several concerns:

1. The only concern which may be considered as major is explanation of the high-intensity band in the silver-stained gel (Figure 1A, line "Final Pellet"). When I compare it to the western blot gel (Fig. 1B), it's quite clear that the top band in "Final Pellet" should correspond to di-glycosylated PrP, which is known as PrP27-30 in literature, a bit lower weak band is for mono-glycosylated PrP, while the third band should be non-glycosylated PrP. But there is a major band at the very bottom of the gel, which has no corresponding band in the western blot. If it can't be explained as some kind of artifact, then identification of what protein or peptide corresponds to this band is crucial for the whole study, as this protein/peptide seems to be the major component of the final pellet.

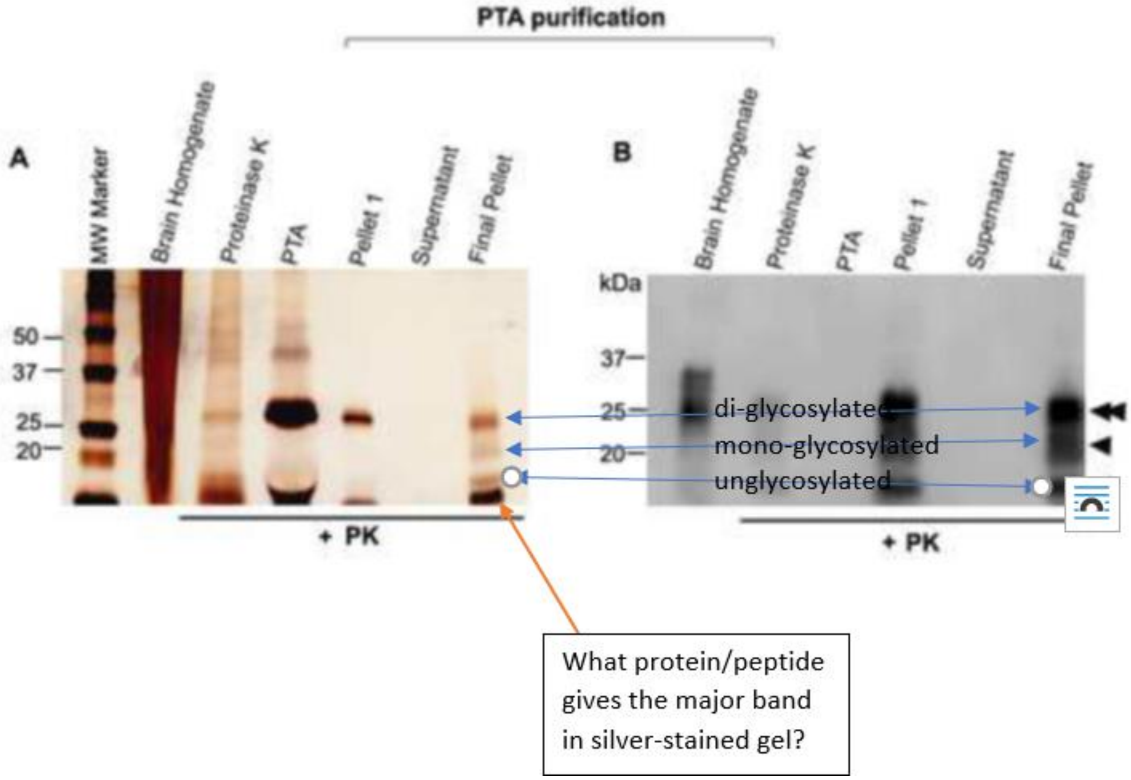

2. The authors state "*Moreover, the presence of two distinct and separable protofilaments in these images contradicts the PIRIBS model, which proposed an in-register stacking of PrPSc monomers covering the full width of a "two protofilament" structure*" and "*From the two main models for prion structure, our findings are compatible with the β-solenoid*

model only, as we identified both one- and two-protofilament fibrils in our samples of purified L-type BSE prions". I can't agree that these statements are valid, as the one-protofilament L-type BSE fibril has similar thickness as two-protofilament fibril, which was used to propose PIRIBS model. From the studies of other proteins, which can form amyloid fibrils we know that morphology of fibrils may change with time (Adamcik, J., Jung, J.-M., Flakowski, J., De Los Rios, P., Dietler, G., and Mezzenga, R. (2010). Understanding amyloid aggregation by statistical analysis of atomic force microscopy images. Nature Nanotechnology 5, 423–428. ; Adamcik, J., Castelletto, V., Bolisetty, S., Hamley, I.W., and Mezzenga, R. (2011). Direct observation of time-resolved polymorphic states in the self-assembly of end-capped heptapeptides. Angewandte Chemie - International Edition 50, 5495–5498. ; Usov, I., Adamcik, J., and Mezzenga, R. (2013). Polymorphism Complexity and Handedness Inversion in Serum Albumin Amyloid Fibrils. ACS Nano 7, 10465–10474.,) for example thin protofilaments, twisted together, after longer incubation may look like one thicker filament. I think that brain-derived material consists mostly of mature fibrils, so one-protofilament fibril may in fact contain two protofilaments, just we have not enough resolution to detect it.

3. Finally, I think the statement in results section "*The mass difference between these two prion variants can easily explain the apparent difference fibril widths*" contradicts with the information in discussion section "*The maximum fibril width for bovine peptide fibrils was found to be 17.0 nm (49), which is close to the width of our two-protofilaments fibrils of bovine PrP 27-30 prions*."

Best Regards,

Vytautas Smirnovas
