# Supplementary material for: The ultrastructure of infectious L-type bovine spongiform encephalopathy prions constrains molecular models
Source: PLoS Pathog. 2021 Jun 1;17(6):e1009628. doi: 10.1371/journal.ppat.1009628 (PMC8195424; doi:10.1371/journal.ppat.1009628)
Supplement: Attachment — Submitted filename: Response to Reviewers.pdf [file ppat.1009628.s003.pdf]

We would like to thank the reviewers for their insightful comments and critiques. We have addressed those criticisms point-by-point in this 'response to reviewers' letter and through the changes we made in the manuscript.

Reviewer #1:

Part I

This intriguing study from a foremost group in this area provides EM-based morphological analyses of L-BSE prions produced in bovinized mice. Interestingly, two fibril morphologies were seen, containing either one or two protofilaments, with the one-protofilament fibrils being more common. This work should make a valuable contribution to the field. However, I have concerns that should be addressed about the characterization of the fibril preparations as well as the authors' interpretations of their data.

Part II

1. The staining in the gradient fraction lanes is too weak to be useful, i.e., almost invisible.

We provided a new silver stained gel for Figure 1C, which now shows the sucrose step gradient fractions, particularly the pellet wash fraction, more clearly.

2. How do these purified bands compare to L-BSE fibrils prepared from cattle brain? Side-by-side lanes should be provided for bovine- and mouse-derived preps, if possible. At the very least, appropriate comparisons to gel data for L-BSE in the literature should be made. What, if anything, has happened to the dark silver-stained band in the "final pellet in panel A, and "pellet 1" in panel C, on the subsequent sucrose gradient? Most importantly, can the authors provide evidence for or against the possibility that with passage of L-BSE into bovinized mice that another strain that might have arisen that accounts for either the single or double protofilament ultrastructures in their preparations? Such prion strain alterations are more likely to occur during interspecies transmissions.

We added a new Western blot panel (Figure 1E) showing a comparison of the migration patterns for the L-type BSE prions with samples containing classical (C-type) and H-type BSE prions that were propagated in the same transgenic mouse line (Tg4092). We performed this experiment to analyze and confirm the strain-specific features of our purified L-type BSE samples. As indicated in previous reports, L-type BSE prions are characterized by a slightly lower position of the unglycosylated, PK-resistant band seen in Western blots. Consistent with those reports, our Western blot analysis (Figure 1E) shows a slightly lower position for the unglycosylated band of the PK-treated PrP 27-30.

The dark ~16 kDa band in the silver stained gels (Figure 1A / 1C) was found to contain proteolytic fragments of various proteins that co-purify with PrP 27-30 when using this PTA-

purification method (Wille et al., 2009; PNAS 106: 3740-3745). The sucrose step gradient removes this band / the contaminating peptides efficiently (see Fig 1C), which is one of the reasons why we included this step in our purification procedure.

Lastly, since the Tg4092 mice express bovine PrP on a murine PrP knock-out background, these mice were found to "faithfully reproduce the hallmarks of the bovine disease" (Scott et al., 1997; PNAS 94: 14279-14284).

Part III

1. The authors state in multiple places, such as line 201-2, that single protofilament prion fibrils have not been reported previously, but this is not true. See, for example, Sim & Caughey, Neurobiol. Aging 2009. Thus, the authors need to remove such claims of novelty.

As requested, we modified these statements in the manuscript and claims of novelty were removed.

2. L218-219: "Moreover, the presence of two distinct and separable protofilaments in these images contradicts the PIRIBS model…" This is not true because two high resolution cryo-EM structures of synthetic PrP fibrils with PIRIBS architectures feature two protofilaments. See C. Glynn et al., Nat. Struct. Mol. Biol., 2020 & L. Q. Wang et al., Nat Struct Mol Biol 2020.

We have removed this statement.

3. L221-3: "These micrographs preclude the possibility that stain artifacts contribute to the observed gap between the protofilaments, as the protofilaments separate into distinct fibrils." This statement should be softened because such results could also be obtained if PK cleaved an exposed chain that links two domains of a PIRIBS-based fibril. For unequivocal evidence that distinct N- and C-terminal domains can exist in infectious prion fibrils, see https://www.biorxiv.org/content/10.1101/2021.02.14.431014v1.

We have rewritten the statement to better fit our experimental observations and softened our interpretation.

4. Indeed, although the authors would not have known about this recently posted high-resolution prion structure in bioRxiv prior to submission of their manuscript, I would encourage them to adjust their interpretations and conclusions during revision with the bioRxiv data in mind (see below). I would argue that broadening their conclusions to include the possibility of PIRIBS-

based architectures for L-BSE fibrils will improve, rather than detract from, the strength of their findings.

We included the results of the high-resolution cryo-EM structure described in the bioRxiv pre-print for our interpretations, discussions, and conclusions and adjusted these texts accordingly. We also used the cross-section of the PIRIBS structure in our comparison of fibril cross-sections and protein structures / models (Fig 10) (see below).

5. L290-2: “Superimposition of the recent 4RβS model combined with N-linked glycans (24, 25) with cross-sections from our 3D reconstructions showed that these reconstructions fit well with the 4RβS model (Fig 10).” Certainly the 4RβS model fits within the cross-section, but it seems to be much too small to fill the space.

Several factors contributed to the apparent mismatch in size between the 4RβS model and the fibril cross-section, which are now described in more detail. In brief, the glycans for the 4RβS model underrepresented their true size, the GPI-anchor was not included, and the model structure was added in without the hydration shell that surrounds all proteins. Overall, the reconstructions are quite compatible with the 4RβS model.

Now, with the new cryo-EM structure, we wanted to see if our reconstructions of L-type BSE fibrils are compatible with the reported high-resolution PIRIBs structure. As shown in the new Figure 10 E panel, we superimposed our cross sections of one- and two-protofilament L-type BSE fibrils with the PIRIBS structure. The overlay of the L-type BSE reconstructions still appears to be more compatible with the 4RβS model than with the PIRIBS structure. However, without a high-resolution structure for the BSE prions, it is not possible to confirm or exclude a particular model or structure.

6. L376-7: “An abundance of experimental evidence indicates that the core structure of the infectious prion contains a 4RßS fold”. This is stated too strongly, especially given the recent posting of a high-resolution prion structure with a PIRIBS architecture (see bioRxiv link above). I would suggest that “indicates” be replaced with “is compatible with”.

Thank you for your suggestion. We replaced “indicates” with “is compatible with”.

7. L385-387: “From the two main models for prion structure, our findings are compatible with the ß-solenoid model only as we identified both one- and two-protofilament fibrils in our samples of purified L-type BSE prions”. I would strongly caution against such a categorical statement. First, as referenced above, PrP fibrils with PIRIBS architectures have been shown with high resolution to be able to have one- or two-protofilaments. Second, the authors seem to

be implying that the size of their single-protofilament L-BSE fibril cores (i.e. averaging 10.6 nm) are too small to be compatible with a PIRIBS architecture. For example, in the Abstract they conclude that “The fact that the one protofilament fibrils contain both N- and C-terminal PrP epitopes constrains molecular models for the structure of the infectious conformer in favour of a compact four-rung β-solenoid fold and excludes more extended fold models.” However, the ordered PIRIBS-based core of 263K scrapie prion fibrils is ~4 x 13 nm (see bioRxiv link above), and one can readily imagine that plausible strain-dependent permutations in this structure could reduce the largest dimension of the core by a mere 1.4 nm, while still maintaining an extended chain PIRIBS-based architecture.

We have refined our arguments and interpretations and softened our statements accordingly.

Reviewer #2:

Part I:

1. The EM data is certainly interesting, as are the single and double protofilament arrangements of the rodent-passaged BSE prions. However, the main point made seems to be that this data only supports a 4RbS model, and this is not sufficiently supported by the presented data. The resolution of the reconstructions is insufficient to conclude the fibrils are formed by 4RbS (or any other model). This study also relies on negatively stained fibrils, which helps with visualization of the fibrils, but could conceivably introduce artifacts into the imaging data and subsequent reconstruction analyses related to the expected variabilities of negative stain densities.

With the release of the pre-print for the 263K prion structure (Kraus et al., 2021; bioRxiv), which was found to adopt a PIRIBS configuration, we softened our statements throughout the manuscript. We also added more explanations in support of our interpretations.

2. The observation of one versus two protofilaments is used as direct evidence to argue for a 4RbS molecular model and most directly against PIRIBS. The authors don't address that there are many examples of two protofilament fibrils that are formed from two parallel, in register architectures including examples for tau, aSyn, and prion protein fragments.

We have refined our interpretations and removed the statements claiming that our results exclude a specific structure.

3. Further, the cross-section of a single 4RbS looks to be much smaller than the density observed in the single BSE protofilament. If a 4RbS, one might even expect multiple 4bS within the single

protofilament width described here, especially as it's unclear if glycans and lipid anchors would be resolved after 2D class averages and 3D reconstructions.

As mentioned in response to reviewer 1 (see above) the 4RβS model contains only small glycans that underrepresent their true size. Both glycans and the GPI-anchor would contribute to the overall diameter of the fibrils, even if they cannot be resolved individually. Moreover, the protein model is shown without the hydration shell of the protein, which would add another layer of density. Therefore, we believe that the 4RβS model is, in fact, a good fit for the fibril cross-sections.

4. Neither the reconstructed topologies, or the Fab data (without further biochemical evidence of what amino acid/structural elements are found at the interface between the two protofilaments) convincingly support or exclude any molecular model (4RbS, PIRIBS, or otherwise).

At the resolution that is achievable with negative stain electron microscopy, the Fab data were not intended to provide precise localizations of the corresponding epitopes. However, the Fab data indicate that the narrow one-protofilament fibrils contain both N- and C-terminal epitopes of the PrP 27-30 molecules. Moreover, the apparent transitions between the one- and two-protofilament morphologies (Figure 4) suggest that the two-protofilament fibrils may contain two distinct fibrillar assemblies. However, we acknowledge that a low-resolution EM study cannot exclude any molecular model. Therefore, we adjusted the interpretations of our results and the subsequent discussions accordingly.

5. Very recently posted descriptions suggest that at least one mammalian prion is a piribs (with fibrils of comparable widths to those noted here for the single BSE protofilament). The authors might consider if alternative models to a single 4RbS per filament are also compatible within their EM data.

As mentioned in response to reviewer 1 (see above), we included a discussion of the newly described PIRIBS structure into our manuscript. In particular, we also included an overlay of the PIRIBS structure with our fibril cross sections (Figure 10E), which provides a better basis for a comparison of the PIRIBS structure and the 4RβS model with the cross sections.

Part III

1. Figure 1 figures are cropped very close to the bands of interest. The authors should show an expanded image of the blots/gels.

As requested, we expanded the images of the Western blots and silver stained gels in Figure 1.

2. How are the crossover distances being defined and determined for the two protofilament fibrils? At least in the micrographs shown it seems sometimes variable - an example micrograph with crossovers indicated would be helpful.

As requested by the reviewer, we indicated some crossover positions in two of the electron micrographs in Figure 3A.

As mentioned in the manuscript, the crossover distances were determined manually in fibrils showing clear periodicities with regard to the stain-filled gap. However, the distances between crossovers in the two-protofilament fibrils were variable, and the value we provided is the average crossover distance. It is now stated within the text that the values refer to the average crossover distance of the fibrils.

3. In Methods, pixels are used to describe the box size used in particle selection and reconstructions. What is the Å/pixel conversion? Imaging magnification?

Micrographs used for 3D reconstructions were recorded at a magnification of 29,000x, with a pixel size of 3.07 Å per pixel. We have now included this information in the manuscript.

4. The immunogold labelling appears to also label amorphous material in addition to the fibrils. Is this a frequent observation?

While in our immunogold labeling experiments the majority of fibrillar assemblies were labeled, labeling of some amorphous aggregates was also observed. We assume that the amorphous material contains non-fibrillar assemblies of PrP 27-30 as had been described earlier (Wille et al., 2009; PNAS 106: 3740-3745).

Reviewer #3:

Part I

The manuscript describes structural studies of a specific L-type strain of infectious prions. Structural studies of brain-derived prions are extremely complicated, so our knowledge on the structure of infectious prions is still limited and each new piece of information is important. So, I strongly support publication of this paper in PLoS Pathogens.
The experiments are well-executed, and the paper is well-written, so I have just several concerns

Part II

1.The only concern which may be considered as major is explanation of the high-intensity band in the silver-stained gel (Figure 1A, line “Final Pellet”). When I compare it to the western blot gel (Fig. 1B), it’s quite clear that the top band in “Final Pellet” should correspond to di-glycosylated PrP, which is known as PrP27-30 in literature, a bit lower weak band is for mono-glycosylated PrP, while the third band should be non-glycosylated PrP. But there is a major band at the very bottom of the gel, which has no corresponding band in the western blot. If it can’t be explained as some kind of artifact, then identification of what protein or peptide corresponds to this band is crucial for the whole study, as this protein/peptide seems to be the major component of the final pellet.

As mentioned in response to reviewer 1 (see above), the ~16 kDa band that can be seen in silver-stained gel sample of the “Final Pellet” (Figure 1A) contains mixture of proteolytic fragments that co-purify with PrP 27-30 during the PTA purification procedure (Wille et al., 2009; PNAS 106: 3740-3745). The sucrose step-gradient centrifugation removes these peptides (compare Figure 1C), which is why these samples were used for our electron microscopy analyses.

Part III

1.The authors state “Moreover, the presence of two distinct and separable protofilaments in these images contradicts the PIRIBS model, which proposed an in-register stacking of PrPSc monomers covering the full width of a “two protofilament” structure” and “From the two main models for prion structure, our findings are compatible with the β-solenoid model only, as we identified both one- and two-protofilament fibrils in our samples of purified L-type BSE prions”. I can’t agree that these statements are valid, as the one-protofilament L-type BSE fibril has similar thickness as two-protofilament fibril, which was used to propose PIRIBS model. From the studies of other proteins, which can form amyloid fibrils we know that morphology of fibrils may change with time (Adamcik, J., Jung, J.-M., Flakowski, J., De Los Rios, P., Dietler, G., and Mezzenga, R. (2010). Understanding amyloid aggregation by statistical analysis of atomic force microscopy images. Nature Nanotechnology 5, 423–428. ; Adamcik, J., Castelletto, V., Bolisetty, S., Hamley, I.W., and Mezzenga, R. (2011). Direct observation of time-resolved polymorphic states in the self-assembly of end-capped heptapeptides. Angewandte Chemie - International Edition 50, 5495–5498. ; Usov, I., Adamcik, J., and Mezzenga, R. (2013). Polymorphism Complexity and Handedness Inversion in Serum Albumin Amyloid Fibrils. ACS Nano 7, 10465–10474.,) for example thin protofilaments, twisted together, after longer incubation may look like one thicker filament. I think that brain-derived material consists mostly of mature fibrils, so one-protofilament fibril may in fact contain two protofilaments, just we have not enough resolution to detect it.

The overall diameters of the one- and two-protofilament fibrils are significantly different $p < 0.0001$ (see Figure 5). Also, the observation of apparent transitions between one- and two-protofilament fibrils (Figure 4) make us confident that we can distinguish these two morphologies in our electron micrographs. We expanded the comparison of the fibril cross-sections to include the PIRIBS structure in addition to the 4RβS model (Figure 10E). We think that this new side-by-side comparison is still in favour of the 4RβS model, but have softened our wording as the resolution of our reconstructions is indeed limited.

2. Finally, I think the statement in results section "The mass difference between these two prion variants can easily explain the apparent difference fibril widths" contradicts with the information in discussion section "The maximum fibril width for bovine peptide fibrils was found to be 17.0 nm (49), which is close to the width of our two-protofilaments fibrils of bovine PrP 27-30 prions."

We removed the section mentioning the bovine PrP 127-147 peptide as it is not directly relevant to the work that is described in this manuscript.
